# WinBEST-KIT: Biochemical Reaction Simulator for Analyzing Multi-Layered Metabolic Pathways

**DOI:** 10.3390/bioengineering8080114

**Published:** 2021-08-11

**Authors:** Tatsuya Sekiguchi, Hiroyuki Hamada, Masahiro Okamoto

**Affiliations:** 1 Department of Life Sciences and Informatics, Faculty of Engineering, Maebashi Institute of Technology, 460-1, Kamisatori-cho, Maebashi 371-0816, Japan; 2 Department of Bioscience and Biotechnology, Faculty of Agriculture, Kyushu University, 744, Motooka, Nishi-ku, Fukuoka 819-0395, Japan; hamada@brs.kyushu-u.ac.jp; 3 Graduate School of Systems Life Sciences, Kyushu University, 744, Motooka, Nishi-ku, Fukuoka 819-0395, Japan; okahon@kyudai.jp

**Keywords:** biochemical reaction simulator, multi-layerd metabolic pathways, systems biology, SBML

## Abstract

We previously developed the biochemical reaction simulator WinBEST-KIT. In recent years, research interest has shifted from analysis of individual biochemical reactions to analysis of metabolic pathways as systems. These large-scale and complicated metabolic pathways can be considered as characteristic multi-layered structures, which, for convenience, are separated from whole biological systems according to their specific roles. These pathways include reactants having the same name but with unique stoichiometric coefficients arranged across many different places and connected between arbitrary layers. Accordingly, in this study, we have developed a new version of WinBEST-KIT that allows users (1) to utilize shortcut symbols that can be arranged with multiple reactants having the same name but with unique stoichiometric coefficients, thereby providing a layout that is similar to metabolic pathways depicted in biochemical textbooks; (2) to create layers that divide large-scale and complicated metabolic pathways according to their specific roles; (3) to connect the layers by using shortcut symbols; and (4) to analyze the interactions between these layers. These new and existing features allow users to create and analyze such multi-layered metabolic pathways efficiently. Furthermore, WinBEST-KIT supports SBML, making it possible for users to utilize these new and existing features to create and publish SBML models.

## 1. Introduction

With recent progress in molecular biology, understanding of the molecular mechanisms of individual biochemical reactions has advanced at a rapid pace. However, static knowledge on the molecular level does not provide insights into the functional properties of whole biological systems, and, thus, research interest has shifted from analysis of individual biochemical reactions to analysis of metabolic pathways as systems. For that reason, systems biology [1,2], which aims to understand interactions among many biomolecules as a system, has become widely accepted. Mathematical models are powerful tools for analysis in systems biology. Rapid improvements in computing power in recent years have made it possible to solve large-scale and complicated mathematical models that were previously intractable [3]. However, it remains very difficult for experimental researchers to describe and simulate corresponding mathematical models using cumbersome programming languages, even when useful models have already been published. For that reason, biochemical reaction simulators that can be used without a deep understanding of mathematical models or information science are being actively developed, as typified by CellDesigner [4,5], COPASI [6,7,8], Cell Illustrator [9,10], DBSolve Optimum [11], BioUML [12,13], and iBioSim [14,15]. The development of biochemical reaction simulators has become one of the most important research topics in systems biology [16]. Furthermore, systems biology markup language (SBML) has been formulated by Hucka et al. as a standardized file format for exchanging models describing biochemical reaction networks and for collaboration using these simulators [17,18].

Accordingly, we previously developed the biochemical reaction simulator Windows-based Biochemical Engineering System analyzing Tool-KIT (WinBEST-KIT) [19,20]. One particularly notable feature is that users can define kinetic equations as user-defined symbols and customize them into the diagrammed modeling interface. The customized symbols represent approximations of unknown kinetic mechanisms; and thus, users can visually arrange their kinetic equations anywhere in the editing area at anytime. For example, Shinto et al. used this feature to create and analyze a kinetic mathematical model for acetone-butanol-ethanol fermentation in *Clostridium saccharoperbutylacetonicum* N1-4 (ATCC13564), which is known as a large-scale and complicated metabolic pathway [21,22]. Sakata et al. also utilized this feature to create and analyze two kinetic mathematical models of soybean metabolic pathways, namely control and flooded models, based on metabolic profiles in soybean plants [23]. Furthermore, we have implemented features of SBML in a way that takes full advantage of particularly notable feature of WinBEST-KIT, as mentioned earlier. Users can also define algebraic equations (AssignmentRule) and events, the characteristic features of SBML, as user-defined symbols and customize them into the diagrammed modeling interface, through the same interface as used in the definition and customization of kinetic equations. The customized symbols can represent the calculation of values based on algebraic rules and the occurrence of events such as resetting parameters when a specific trigger equation is satisfied. The details for defining and customizing kinetic equations, algebraic equations, and events as user-defined symbols and a comparison of WinBEST-KIT with the aforementioned simulators are described elsewhere [19,20].

The problem considered here is that, owing to the increase in the scale and complexity of metabolic pathways analyzed in recent years, creating models using a diagrammed modeling interface involving many symbols becomes progressively more difficult as a consequence of the inevitable poor visibility that this scale and complexity causes. Furthermore, these large-scale and complicated metabolic pathways can be considered as characteristic multi-layered structures, which, for convenience, are separated from whole biological systems according to their specific roles. These pathways include reactants having the same name but with unique stoichiometric coefficients arranged across many different places and connected between arbitrary layers. Accordingly, in this study, we have developed a new version of WinBEST-KIT that allows users (1) to utilize shortcut symbols that can be arranged with multiple reactants having the same name but with unique stoichiometric coefficients, thereby providing a layout that is similar to metabolic pathways depicted in biochemical textbooks; (2) to create layers that divide large-scale and complicated metabolic pathways according to their specific roles; (3) to connect the layers by using shortcut symbols; and (4) to analyze the interactions between these layers. These new and existing features allow users to create and analyze such multi-layered metabolic pathways efficiently. Furthermore, WinBEST-KIT supports SBML, making it possible for users to utilize these new and existing features to create and publish SBML models.

## 2. Overview of WinBEST-KIT

**Figure 1:**
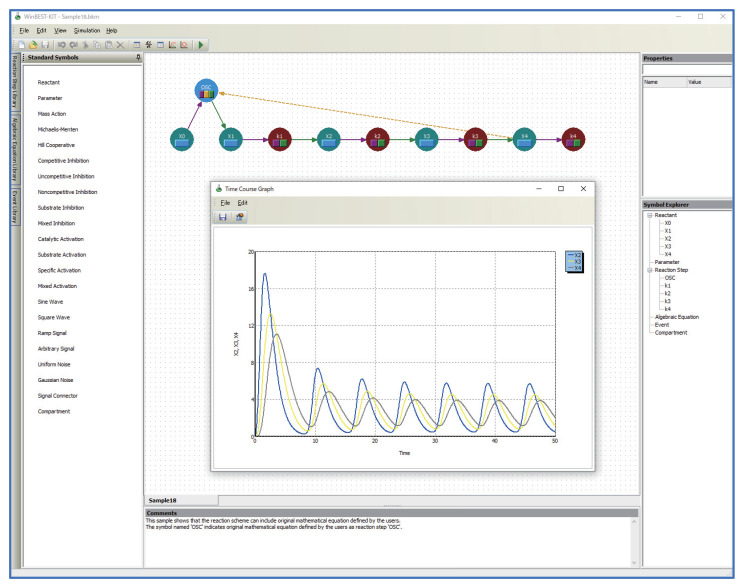
Screenshot of WinBEST-KIT.

**Figure 2:**
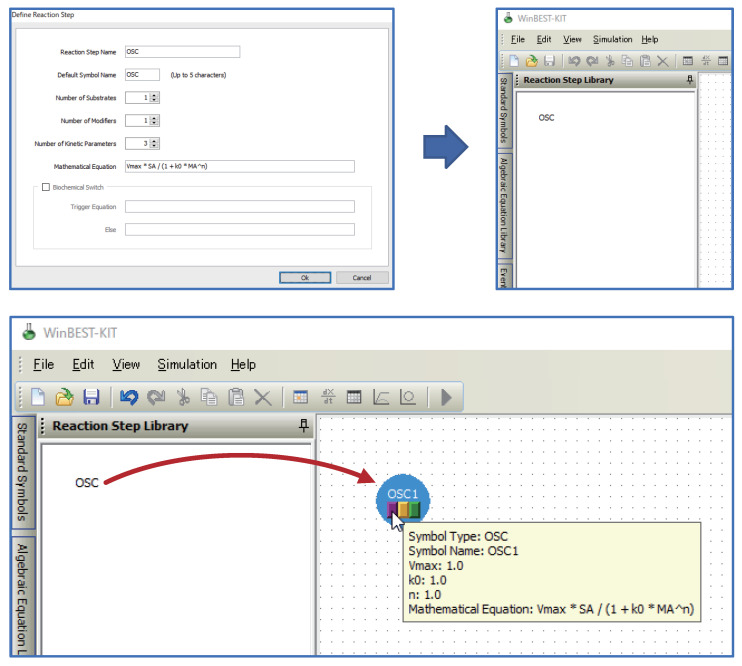
The kinetic equation labeled “OSC” can be registered in the symbol selection area in WinBEST-KIT.

WinBEST-KIT is a Windows application that provides an integrated simulation environment for creating and analyzing large-scale and complicated metabolic pathways. Figure 1 shows a screenshot of WinBEST-KIT. In WinBEST-KIT, the metabolic pathways to be analyzed are represented by circular symbols connected with lines. This representation in WinBEST-KIT is called a “reaction scheme”. The only work that this application necessarily requires of users is the creation of the reaction scheme. Derivation of mass balance equations (i.e., kinetic mathematical models), execution of numerical calculations (i.e., simulation), and visualization of simulation results are all performed automatically based on the created reaction schemes. The features of WinBEST-KIT are as follows:

1. Reaction schemes can be created by operations that are similar to using a drawing tool.
  A reaction scheme representing metabolic pathways can be created by connecting reactant symbols that indicate reactants and reaction step symbols that indicate reaction steps with lines. A wide variety of editing functions (cut, copy, paste, undo, and so on) are also provided;
2. Reaction schemes can be created that contain a combination of mass action law and steady-state approximations of enzyme kinetics.
  Reaction schemes can be created that combine detailed models based on the mass action law along with the steady-state approximations of enzyme kinetics such as the Michaelis–Menten equation. The major steady-state approximations of enzyme kinetics are provided as standard reaction step symbols;
3. Kinetic equations can be used as user-defined symbols for representing unknown kinetic mechanisms.
  Users can define kinetic equations as user-defined symbols and customize them into the diagrammed modeling interface; that is, the custom reaction step symbols can be used in WinBEST-KIT. This feature is called “Reaction Step Library.” Figure 2 shows an example of defining and customizing the kinetic equations. In Figure 2, a kinetic equation labeled “OSC” is defined and customized into the symbol selection area; thus, users can visually arrange any number of “OSC” reaction step symbols onto the editing area anytime and anywhere. Furthermore, the kinetic equations can also be approximated a part of metabolic pathways. Thus, “Reaction Step Library” can also be used as compartmentalization of biochemical reactions;
4. Algebraic equations (AssignmentRule) and events, the characteristic features of SBML, can be used as user-defined symbols.
  Users can define algebraic equations and events as user-defined symbols and customize them into the diagrammed modeling interface through the same interface as used in the definition of the kinetic equations shown in Figure 2; that is, the custom algebraic equation symbols and the custom event symbols can be used in WinBEST-KIT. These features are called “Algebraic Equation Library” and “Event Library,” respectively. Users can visually arrange any number of the custom algebraic equation symbols and the custom event symbols anywhere in the editing area at any time, similar to the custom reaction step symbols;
5. Mass balance equations can be automatically derived, with automatic simulation.
  WinBEST-KIT can automatically derive mass balance equations that represent a created reaction scheme. Because of this, users do not need to be aware of the underlying simultaneous ordinary differential equations. Users also do not need to be aware of the numerical calculation methods or of programming languages because the numerical calculations are also executed automatically;
6. High-speed simulations are performed using a compiler.
  The numerical calculations can be performed using an external compiler (see Section 6). Although the compiler needs to be installed, the cumbersome work of compilation is performed automatically thereafter. If users want to try out WinBEST-KIT, numerical calculations can be performed by the built-in interpreter;
7. A wide variety of analysis functions are available.
  WinBEST-KIT provides a wide variety of analysis functions in addition to regular time-course simulation, including estimation of values of kinetic parameters (using the modified Powell method [24], real-coded genetic algorithms [25], and their hybrid method [26,27]), and real-time simulation through the Virtual Lab feature. Especially, estimation of the values of kinetic parameters is a useful function. It can estimate the values of kinetic parameters from experimentally observed time-course data, even if the reaction schemes include the kinetic equations, the algebraic equations, and the events. The analysis results that can be displayed include time-course graphs, phase-plane diagrams, numerical data, and mass balance equations;
8. Import from and export to SBML models.
  SBML is a standardized file format for exchanging models describing biochemical reaction networks, which has been adopted by about 300 applications. More than 1000 SBML models that have been published are stored in the BioModels Database [28,29,30]. Users can analyze and reuse these already published models and publish their created models. Note that some features of SBML cannot be used in WinBEST-KIT (see Section 7);
9. Export to MATLAB m-files.
  Reaction schemes created in WinBEST-KIT can be exported as MATLAB m-files, which allows users to compare the simulation results with this well-established numerical simulation application and to convert SBML models to MATLAB m-files through WinBEST-KIT. Note that reaction schemes created using functions not supported in MATLAB, such as events in SBML, cannot be exported because the exported MATLAB m-files uses the MATLAB built-in ODE solver.

## 3. Considerations for Multi-Layered Metabolic Pathways

**Figure 3:**
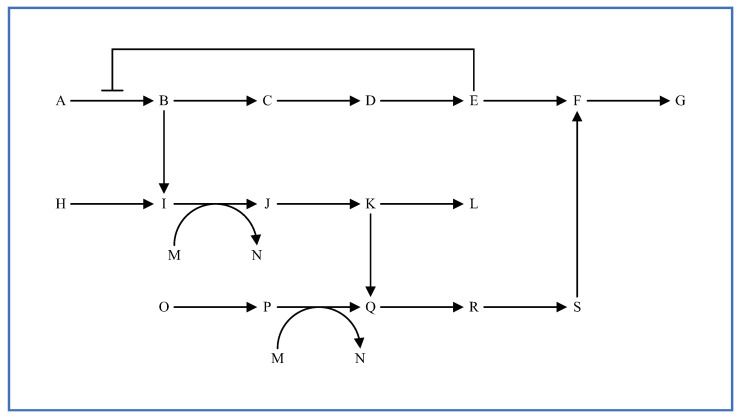
Example metabolic pathway.

**Figure 4:**
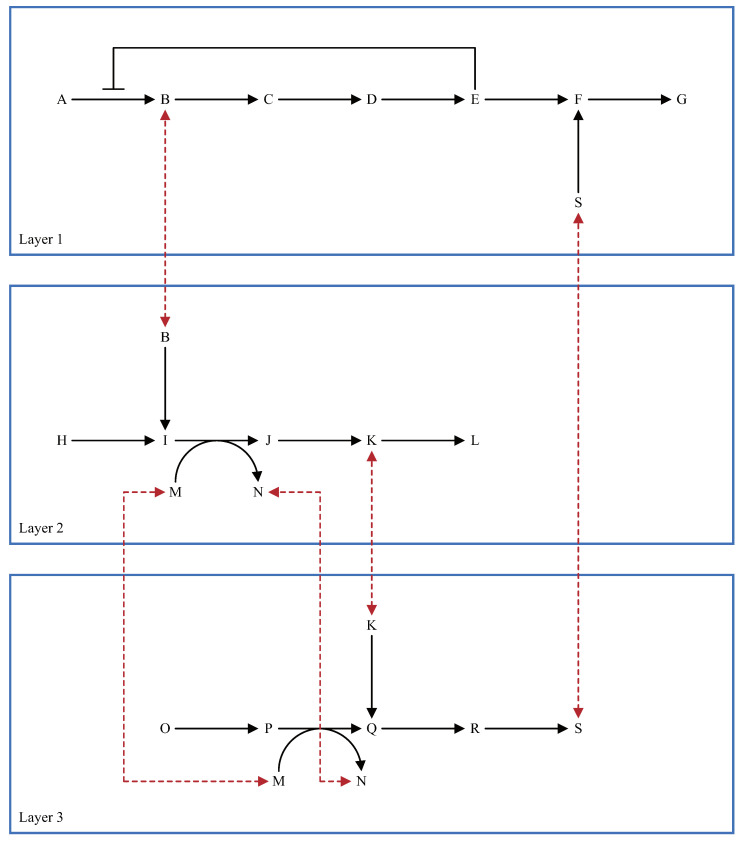
Example of the layer representation of the metabolic pathway shown in Figure 3. The red dashed lines indicate how each layer is connected through the reactants.

Metabolic pathways can be considered as characteristic multi-layered structures. General layered structures have a hierarchical tree structure with upper and lower layers. However, for convenience, the metabolic pathways depicted in biochemical textbooks are separated from whole biological systems according to their specific roles; allowing the reader to divide metabolic pathways as they like. For that reason, there are no higher and lower levels in each divided metabolic pathway. The input reactants and the output reactants as the connectors in each layer can also be decided by users. Each divided metabolic pathway is handled as a “layer” in WinBEST-KIT. Figure 3 shows an example of a metabolic pathway, and Figure 4 shows an example of a layer representation of the metabolic pathway in Figure 3. As shown in Figure 4, we have divided the metabolic pathway in Figure 3 into three layers, with the reactants of some connected to other layers. In this case, it can be considered that layer 1 is connected to layer 2 through reactant B and connected to layer 3 through reactant S. Similarly, layer 2 can be considered as connected to layer 1 through reactant B and layer 3 through reactants K, M, and N. Layer 3 is also connected to layer 1 through reactant S and to layer 2 through reactants K, M, and N. As shown in Figure 4, each layer is simply divided according to its specific roles, and there is no hierarchical tree structure. For that reason, Figure 3 and Figure 4 demonstrate that the connections between the layers in the metabolic pathways have no standard rule. The most important point is that the same reactants are arranged into several layers and that they connect these layers. For example, reactant B in layers 1 and 2 is the same reactant, and it connects layers 1 and 2. As a more practical example, glycolysis and the citric acid cycle can be considered typical layers in the metabolic pathway. Here, ATP, ADP, NAD${}^{+}$, NADH, and pyruvic acid are the reactants that are connecting these two layers.

**Figure 5:**
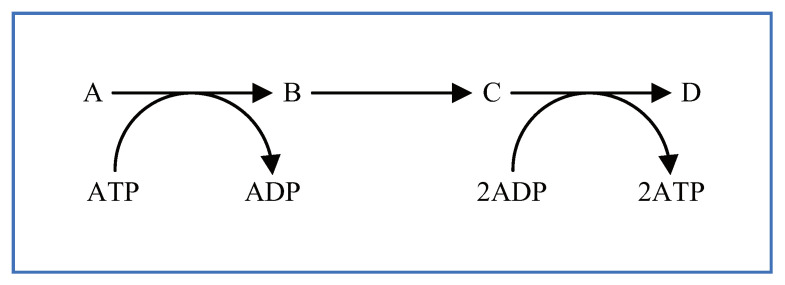
ATP, 2ATP, ADP, and 2ADP appear in the same metabolic pathway.

Additionally, reactants having the same name are arranged across many different places to more clearly represent the metabolic pathways, making them easier to understand. Arranging reactants in this way illustrates that the same reactants are utilized or produced in various reaction mechanisms in the same metabolic pathways. For example, ATP, ADP, NAD${}^{+}$, NADH, and so on, are arranged across many different places in the same metabolic pathway. Furthermore, despite having the same name, their stoichiometric coefficients can differ depending on the reaction mechanisms. Figure 5 shows a typical example of a reaction mechanism that can be seen throughout the metabolic pathways. In Figure 5, ATP and ADP are arranged in the reaction mechanism of the first reaction step and their stoichiometric coefficient is 1. ATP and ADP are also arranged in the reaction mechanism of the third reaction step and their stoichiometric coefficient is 2. As a more practical example, 2ATP and ATP, 2ADP and ADP, 2NAD${}^{+}$ and NAD${}^{+}$, and 2NADH and NADH can be arranged within the glycolysis pathway.

Thus, to represent such multi-layered metabolic pathways in the diagrammed modeling interface in biochemical reaction simulators, it is necessary that metabolic pathways can be divided into arbitrary layers as the user chooses.

## 4. Implementation of New Features for Analyzing Multi-Layered Metabolic Pathways

To overcome these problems as mentioned in Section 3 and to enable the creation and analysis of such multi-layered metabolic pathways, we have implemented new features in WinBEST-KIT.

We have implemented multiple editing areas; and, thus, users can simultaneously create several reaction schemes divided into layers. Each editing area can be switched by clicking on the tabs displayed at the bottom. The pointers to each symbol in the editing area are managed for each editing area. When users save a reaction scheme to a file, the layer information is preserved. At the beginning of the simulation, the pointers of all symbols in all of the editing area (across layers) are assembled. By doing this, all the layers can be treated as a single large-scale reaction scheme. However, when users export the created reaction scheme to SBML models, they are stored as non-layered models because SBML cannot represent such multi-layered information at present; SBML can use compartments, but their role differs from that of the layers in WinBEST-KIT.

We have implemented shortcut symbols for linking corresponding target reactant symbols. Users can create any number of the shortcut symbols in the editing area, even if the corresponding target reactant symbol is arranged in a different layer. Thus, the shortcut symbols in the different layers are connectors between these layers, and users can divide large-scale and complicated metabolic pathways as they choose. Both the reactant symbols and the shortcut symbols have four properties in common: symbol name, concentration, reactant type (variable or pool), and stoichiometric coefficient. Additionally, the shortcut symbols have pointers to their corresponding target reactant symbols. The symbol name, concentration, and reactant type can only be edited in the property editor for the reactant symbols. These three properties of the shortcut symbols are synchronized immediately when these three properties of their corresponding target reactant symbols are updated. However, all reactant symbols and all shortcut symbols must have a unique stoichiometric coefficient for each reaction mechanism involved. For that reason, the stoichiometric coefficient can be edited in the property editor of both the reactant symbols and the shortcut symbols, and a unique value can be set for all reactant symbols and all shortcut symbols. At the derivation of the mass balance equations, a unique ID for the numerical calculation has been assigned to all reactant symbols, but it has never been assigned to the shortcut symbols. Each reaction mechanism refers to the connected reactants, and then, when they are represented by shortcut symbols, they use an ID assigned to their corresponding target reactant symbol and use the stoichiometric coefficient of each shortcut symbol when applying its stoichiometry.

## 5. Examples of New Features of WinBEST-KIT

### 5.1. Arrange Shortcut Symbols with Unique Stoichiometric Coefficients

**Figure 6:**
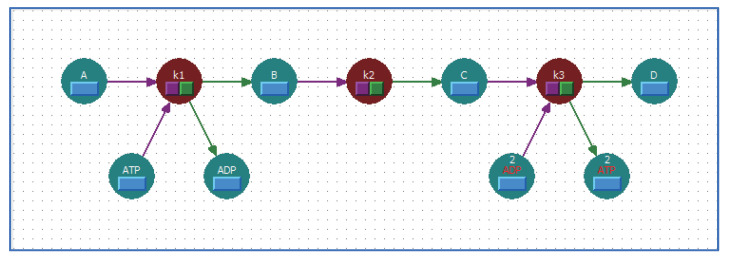
Reaction scheme corresponding to Figure 5 created using WinBEST-KIT.

**Figure 7:**
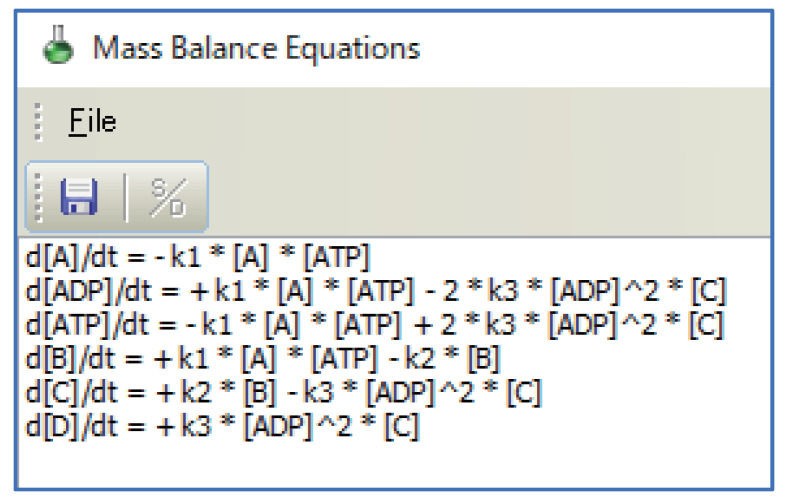
Mass balance equations derived automatically from Figure 6 using a reaction scheme created in WinBEST-KIT.

Consider the creation of a reaction scheme according to the metabolic pathway shown in Figure 5. In this case, one molecule each of ATP and ADP are involved in the first reaction step, and two molecules each of ATP and ADP are involved in the third reaction step. In other words, the number of molecules involved in the reaction mechanism (i.e., the stoichiometric coefficient) differs between the first and third reaction steps. Although the shortcut symbols have synchronized the values for the properties of their corresponding target reactant symbols, the stoichiometric coefficient can be set independently, as mentioned in Section 4. Figure 6 shows the reaction scheme corresponding to Figure 5 created using WinBEST-KIT. In Figure 6, ATP and ADP, arranged in the lower left corner by using the reactant symbols, are involved in the reaction mechanism of the first reaction step; their stoichiometric coefficient is 1. ATP and ADP, arranged in the lower right corner by using the shortcut symbols, with red symbol names, are involved in the reaction mechanism of the third reaction step; their stoichiometric coefficient is 2. The stoichiometric coefficients of each reactant symbol and shortcut symbol are also displayed above the symbol name. Note that such a value is not displayed when the stoichiometric coefficient is 1; see the reactant symbols in the first reaction step. Figure 7 shows the mass balance equations derived automatically from the reaction scheme created in WinBEST-KIT shown in Figure 6. Note that the “^” operator in the mass balance equations indicates exponentiation. As shown in Figure 6 and Figure 7, the stoichiometric coefficients of the shortcut symbols of ATP and ADP involved in the third reaction step have been applied to the derived mass balance equations; thus, reaction schemes with layouts similar to metabolic pathways depicted in biochemical textbooks can be created in WinBEST-KIT.

### 5.2. Multi-Layered Metabolic Pathways

**Figure 8:**
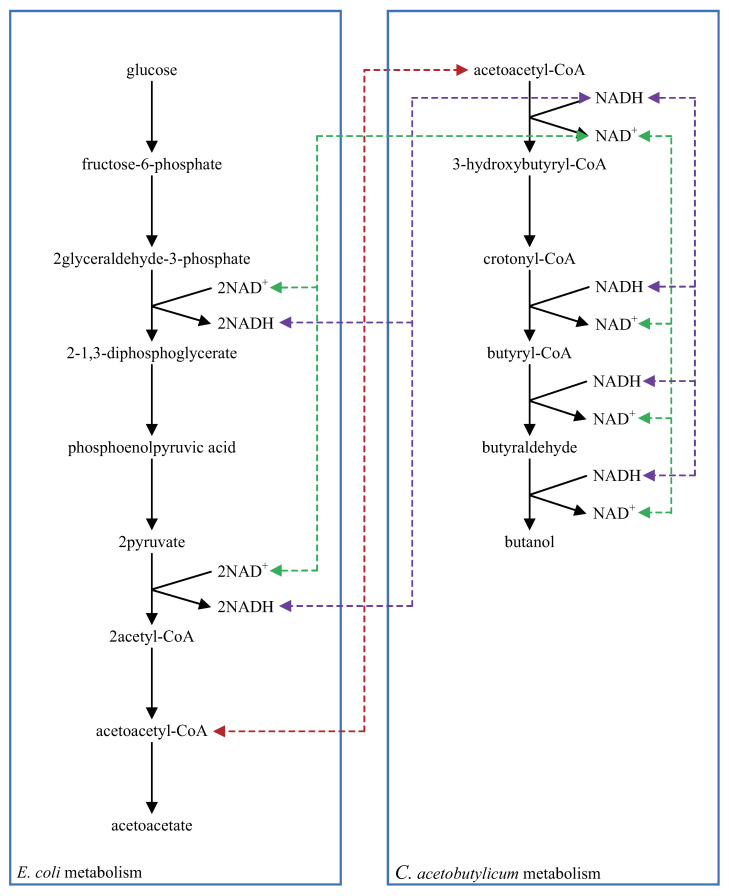
Metabolic pathways of synthetic butanol production using synthetic biology techniques. Acetoacetyl-CoA, NAD${}^{+}$, and NADH with unique stoichiometric coefficients appear in both *Escherichia coli* and *Clostridium acetobutylicum* metabolisms. Dashed lines indicate the connection of these two different metabolisms through these reactants.

**Figure 9:**
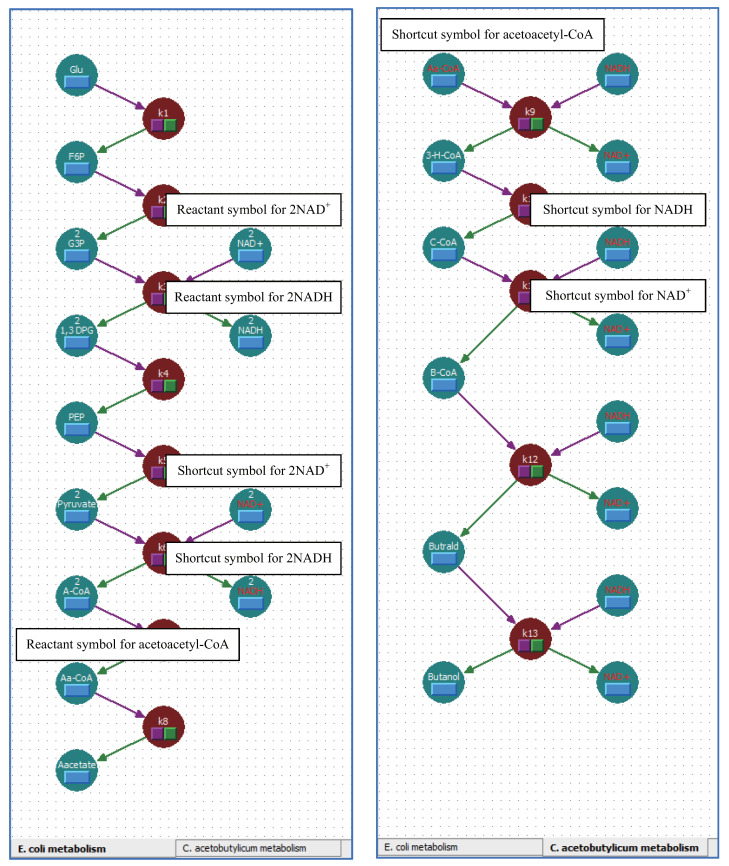
Reaction scheme corresponding to Figure 8 created using WinBEST-KIT. Reactant symbols and shortcut symbols with unique stoichiometric coefficients can be connected between *Escherichia coli* and *Clostridium acetobutylicum* metabolisms in different layers.

Multi-layered metabolic pathways can be created by using shortcut symbols with unique stoichiometric coefficients and connecting layers divided according to their specific roles. Figure 8 shows the metabolic pathways of synthetic butanol production using synthetic biology techniques [31]. Synthetic butanol production consists of two different metabolisms; the left and right sides are *Escherichia coli* and *Clostridium acetobutylicum* metabolisms, respectively. Acetoacetyl-CoA is one of the reactants in both *E. coli* and *C. acetobutylicum* metabolisms. For that reason, these two different metabolisms are connected through acetoacetyl-CoA; consequently, butanol is produced by the interaction between these two different metabolisms. Furthermore, coenzymes NAD${}^{+}$ and NADH also connect these two different metabolisms. The stoichiometric coefficients of NAD${}^{+}$ and NADH have a unique value in each reaction step. The balance of the stoichiometric coefficients of NAD${}^{+}$ and NADH are conserved by these two different metabolisms. Four molecules of NADH are produced in the *E. coli* metabolism, and four molecules of NADH are utilized in the *C. acetobutylicum* metabolism. In contrast, four molecules of NAD${}^{+}$ are utilized in the *E. coli* metabolism, and four molecules of NAD${}^{+}$ are produced in the *C. acetobutylicum* metabolism. The reaction scheme of this complicated metabolism for producing synthetic butanol can be created by using shortcut symbols and layers in WinBEST-KIT. Figure 9 shows the reaction scheme corresponding to Figure 8 created using WinBEST-KIT. These two different metabolisms can be considered as layers connected through the reactant symbols for acetoacetyl-CoA, NAD${}^{+}$, and NADH and their shortcut symbols. These reactant symbols and their shortcut symbols can have the same name, the same concentration, and the same reactant type, but unique stoichiometric coefficients, even if each symbol is arranged on the different layers. For example, at the reaction step of 2glyceraldehyde-3-phosphate to 2-1,3-diphosphoglycerate in the *E. coli* metabolism, two molecules of NADH are produced, and at the reaction step of acetoacetyl-CoA to 3-hydroxybutyryl-CoA in the *C. acetobutylicum* metabolism, one molecule of NADH is utilized. When we create these two reaction steps, the reactant symbol for NADH and its shortcut symbol are arranged, and their stoichiometric coefficients can be set independently as 2 and 1, respectively. Thus, as shown in Figure 9, acetoacetyl-CoA, NAD${}^{+}$, and NADH can be arranged in the same layout as the metabolic pathways shown in Figure 8 by using shortcut symbols and layers.

### 5.3. Applying Shortcut Symbols and Layers to Imported SBML Models

**Figure 10:**
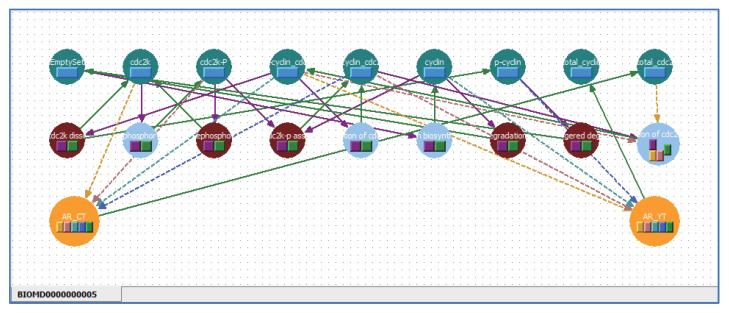
Screenshot immediately after importing a SBML model into WinBEST-KIT. The symbols are arranged according to certain rules because there are no coordinate data in SBML. For that reason, the visibility of the imported SBML models is inevitably poor.

**Figure 11:**
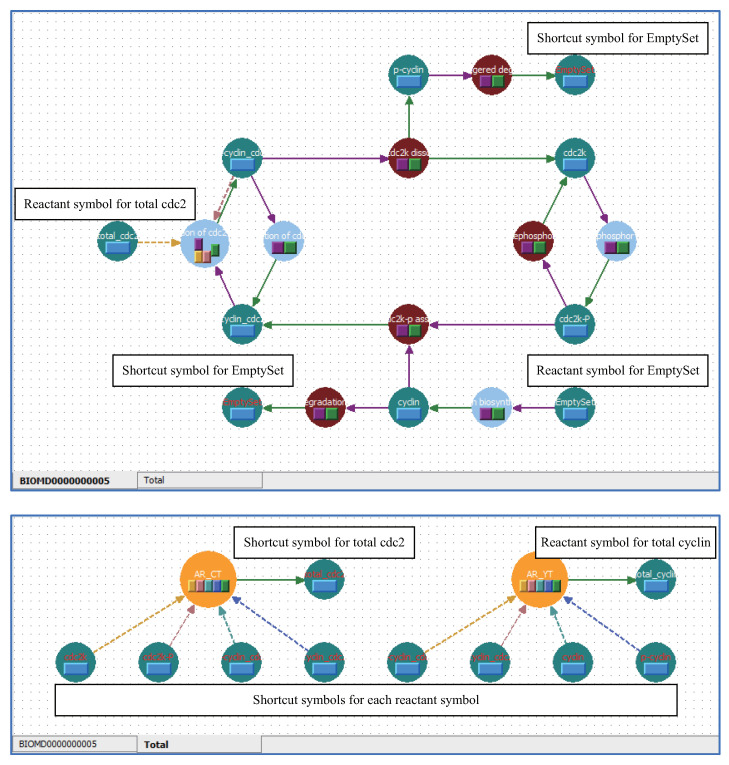
Screenshot after rearranging the symbols. Users can divide the SBML models into layers according to their specific roles, connect the layers, and analyze them as a single integrated model by using the shortcut symbols.

**Figure 12:**
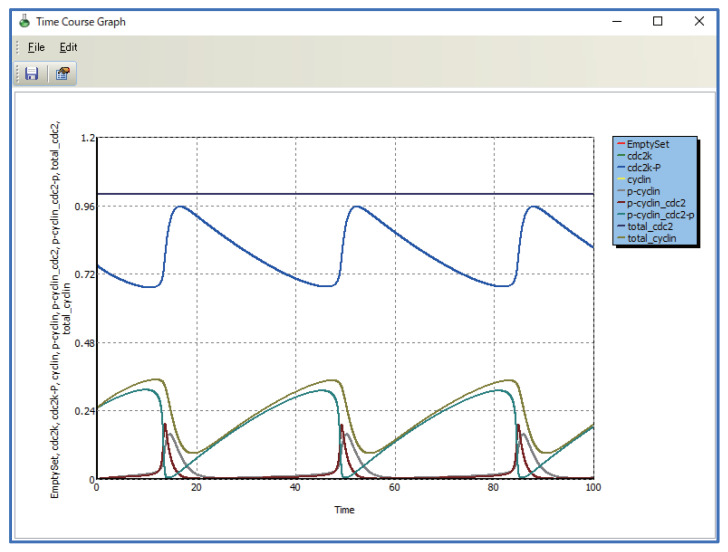
A reaction scheme created from multiple layers can be simulated as a single integrated model, even if it contains algebraic equations.

Complicated models written in SBML can be imported into WinBEST-KIT. The representation of complicated models in the diagrammed modeling interface inevitably has poor visibility. Figure 10 shows a screenshot immediately after importing into WinBEST-KIT the SBML model “BIOMD0000000005_url.xml,” which describes the cell division cycle model published by Tyson (1991) [32] and stored in BioModels Database No. 5 (https://www.ebi.ac.uk/biomodels/BIOMD0000000005, (accessed on 1 August 2021)). As shown in Figure 10, there are many symbols and their connections are complicated, making this model difficult to understand. Other simulators adopting a diagrammed modeling interface have a similar difficulty. This model has two algebraic equations (AssignmentRule) to calculate the total concentration of cyclin and the total concentration of cdc2, respectively. We will arrange these algebraic equations on a different layer to better understand this model more easily through using the shortcut symbols. Furthermore, symbols representing the relationship between cyclin and cdc2 in the cell can be rearranged according to the reference by using shortcut symbols. The ability to visually arrange algebraic equations as symbols is one of the particularly notable features of WinBEST-KIT [20]. Figure 11 shows the created reaction scheme. In the layer labeled “Total” in the lower panel of Figure 11, the algebraic equation (orange) symbol arranged on the left side can be used to calculate the total concentration of cdc2; the four shortcut symbols labeled “cdc2k,” “cdc2k-p,” “p-cyclin_cdc2,” and “p-cyclin_cdc2-p,” are connected as the variables, and the shortcut symbol labeled “total_cdc2” is connected in order to substitute the calculation result (i.e., total_cdc2=cdc2k+cdc2k-p+p-cyclin_cdc2+p-cyclin_cdc2-p). Similarly, the algebraic equation symbol arranged on the right side can be used to calculate the total concentration of cyclin; the four shortcut symbols labeled “cyclin,” “p-cyclin,” “p-cyclin_cdc2,” and “p-cyclin_cdc2-p” are connected as variables, and the reactant symbol labeled “total_cyclin” is connected in order to substitute the calculation result (i.e., total_cyclin=cyclin+p-cyclin+p-cyclin_cdc2+p-cyclin_cdc2-p). It is particularly important that the corresponding target reactant symbol of each shortcut symbol is arranged on a different layer labeled “BIOMD0000000005” (the upper panel of Figure 11). Furthermore, in the layer labeled “BIOMD0000000005,” the reactant symbol labeled “EmptySet” indicates amino acids in the reference; the shortcut symbols make it possible to arrange the multiple EmptySet symbols according to the reference. Figure 12 shows the simulation result of Figure 11, which can obtain the same result simulated using CellDesigner and COPASI. Although the symbols representing the relationship between cyclin and cdc2 in the cell and the algebraic equations for calculating the total concentration of cyclin and the total concentration of cdc2 are arranged on different layers, WinBEST-KIT can treat them as a single integrated model. Thus, calculating the total concentration of cdc2 can be understood to involve the concentration of the four reactants on the different layers, and its result is then used in the calculation of the relationship between cyclin and cdc2 in the cell on a different layer. Accordingly, users can understand complicated models written in SBML easily by using the shortcut symbols and layers in WinBEST-KIT, because users can decide how to divide the models into layers and then analyze the layers as a single integrated model.

## 6. Performance of Computational Time

**Figure 13:**
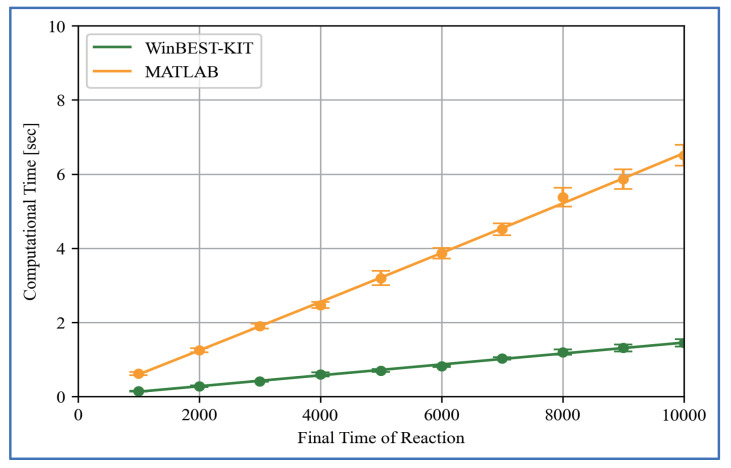
Comparison results of the computational time for the published SBML model as mentioned in Section 5.3. Each measurement point is the mean value of 100 times calculations and the error bar shows its standard deviation.

**Figure 14:**
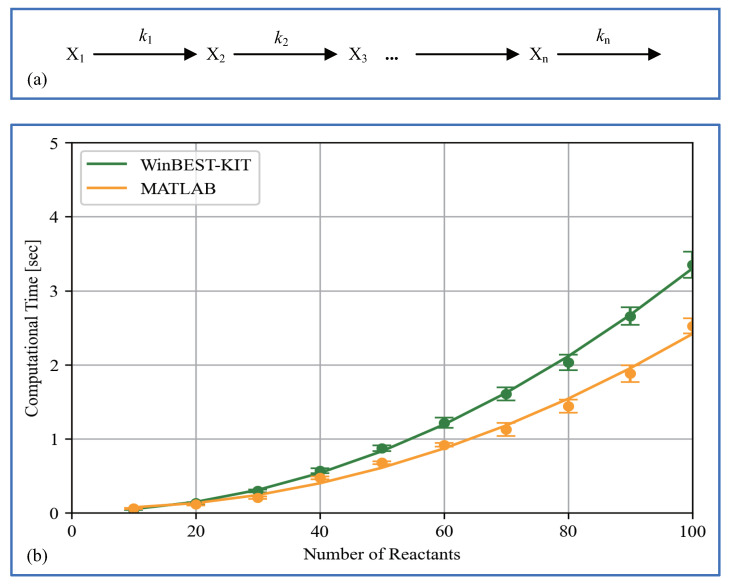
(**a**) System diagram of the linear subsequent reaction system. (**b**) Comparison results of the computational time with respect to the number of reactants. Each measurement point is the mean value of 100 times calculations and the error bar shows its standard deviation.

To solve non-linear simultaneous ordinary differential equations representing large-scale and complicated metabolic pathways, we have implemented an efficient numerical solution method called the Gear method [33] in WinBEST-KIT. Furthermore, the numerical calculations in WinBEST-KIT can be performed faster using an external compiler as mentioned in Section 2 (6). We have examined the computational time in WinBEST-KIT in comparison with those in MATLAB using the published SBML model as mentioned in Section 5.3. The values of kinetic parameters are the same in the reference. The maximum step size of numerical integration is 0.1, which is the default value in WinBEST-KIT. We have measured the computational time with changing the final time of the reaction ($t_{f}$, arbitrary time unit) on a PC (Xeon-1620V4 3.5GHz with 16GB memory). Figure 13 shows the comparison results of the computational time. As shown in Figure 13, even though the final time of the reaction becomes longer, the increase in the computational time is suppressed in WinBEST-KIT; the computational time in WinBEST-KIT have a good performance for this published SBML model. We have also examined the scalability with respect to the number of reactants. Figure 14a shows the linear subsequent reaction system. Fixed the final time of the reaction at 1000 (arbitrary time unit), the computational time have measured with changing the n-value from n=10 to n=100. The initial concentrations of reactants $X_{1}$ and $X_{i}$(i=2,3,…,n) are 100 (constant value) and 0, respectively. The rate constants $k_{j}$(j=1,2,…,n) are 0.1. The maximum step size of numerical integration is 0.1. Figure 14b shows the comparison results of the computational time with respect to the number of reactants, which has measured using the same PC for Figure 13. As shown in Figure 14b, the computational time is slightly faster with MATLAB, and the changes of the scalability is almost same. Thus, as shown in Figure 14, the computational time is slightly faster with MATLAB for the models of simple metabolic pathways, however, as shown in Figure 12 and Figure 13, the numerical calculations in WinBEST-KIT are effective for the models of large-scale and complicated metabolic pathways involving a high non-linearity.

## 7. Future Developments

A problem remaining to be overcome in WinBEST-KIT is compatibility with SBML and multi-platform support. The current version of WinBEST-KIT supports SBML Level 3 Version 1 Core; however, there are a few features of SBML that are not supported at present as the SMBL specification is enormous. Fast reaction, csymbol delay, dynamic change in stoichiometry, and multiple piecewise features as well as a portion of the mathematical functions are not supported in the current version of WinBEST-KIT. Additionally, because WinBEST-KIT uses Windows DLL technology for compiling the derived mass balance equations, WinBEST-KIT cannot be run in macOS or Linux at present.

In the near future, we will adopt LibSBMLSim [34], which is a high-performance numerical simulation engine fully compliant with SBML that has multi-platform, Windows, macOS, and Linux support. Additionally, we will adopt systems biology graphical notation (SBGN) [35,36], which provides a system of standard visual glyphs for systems biology, in order to enhance the visibility of symbols. We will also implement a feature that can retrieve published SBML models from BioModels Database to facilitate analysis. By adopting these features, we aim to increase the corresponding features for SBML and provide support for macOS and Linux.

## 8. Conclusions

Biochemical textbooks often divide large-scale and complicated biological systems into multiple metabolic pathways. However, it is the interactions of these multiple metabolic pathways that make up whole biological systems. In the new version of WinBEST-KIT, layers and shortcut symbols enable users to efficiently create and analyze multi-layered metabolic pathways. Furthermore, WinBEST-KIT supports SBML, making it possible for users to utilize these new and existing features to create and publish SBML models. We anticipate that WinBEST-KIT will provide new insights into the functional properties of whole biological systems.

## Data Availability

The current version of WinBEST-KIT is freely available at http://winbest-kit.org/, accessed on 1 August 2021.
